# Intervention outcome preferences for youth who are out of work and out of school: a qualitative study

**DOI:** 10.1186/s40359-022-00887-5

**Published:** 2022-07-23

**Authors:** Na Zhu, Lisa D. Hawke, Matthew Prebeg, Em Hayes, Karleigh Darnay, Srividya N. Iyer, Joanna Henderson

**Affiliations:** 1 Centre for Addiction and Mental Health, 80 Workman Way, Toronto, ON Canada; 2 Department of Psychiatry, University of Toronto, 250 College Street, Toronto, ON Canada; 3 McGill University, 845 Sherbrooke St W, Montreal, QC Canada; 4 Douglas Hospital Research Centre, 6875 Boulevard LaSalle, Montreal, QC Canada

**Keywords:** Intervention outcomes, NEET, Qualitative, Upcoming Youth

## Abstract

**Background:**

While interventions have been developed and tested to help youth who have become disconnected from work and school, there is a paucity of research on young people’s intervention preferences. This study aims to understand young people’s preferred intervention outcomes and approaches for youth who are out of work and school.

**Methods:**

Thirty youth participated in virtual focus groups. Transcripts were analyzed using thematic analysis.

**Results:**

Youth want interventions and approaches that support them in (1) vocational readiness, (2) securing a job, and (3) mental health and well-being, while providing them with (4) high-contact, individualized, and integrated support.

**Conclusions:**

Young people want interventions to be individualized and integrated, providing a high level of support for their educational and employment pursuits as well as their mental health and well-being. Incorporating youth's perspectives when designing interventions can increase intervention relevance and potentially service uptake, helping youth continue to pursue their educational and vocational goals.

## Introduction

Some young people experience significant difficulties with employment as they transition from education to the workforce[1]. The acronym NEET (Not in Education, Employment, or Training), which first emerged in the United Kingdom in the 1990s, describes a heterogeneous group of individuals who are not engaged in education, employment, or training structures [2]. Given that NEET reflects a deficit-based notion and defines young people by what they are not [3], we use the term ‘Upcoming Youth,’ which was coined by youth stakeholders through the Youth Engagement Initiative at the Centre for Addiction and Mental Health.

The concept of being “NEET”, or an Upcoming Youth, is established in labour market statistics. Across the Organization for Economic Cooperation and Development (OECD), between 5.5 and 28.8% of youth aged 15–29 were out of work and out of school in 2019, at an average of 13.0% [4]. In Canada, 11.3% of young people in 2019 were Upcoming Youth [4]. Among youth-serving organizations in Canada, across the mental health, substance use, education, justice, and housing sectors, over a quarter of service-seeking young people are Upcoming Youth [5].

There is a bi-directional relationship between Upcoming Youth status and psychosocial challenges: many adversity factors increase the risk of being unable to engage in school and work, while being disengaged, in turn, increases the risk of psychosocial challenges [6]. Factors that increase the risk of becoming an Upcoming Youth include having preexisting mental health and substance use problems, suicidality, justice involvement, poor vocational history, low academic achievement, early parenthood, precarious housing, and low socioeconomic status [3, 5, 7–10]. In turn, being unable to engage vocationally increases the risk of social exclusion, poor career prospects and attainment, and other negative psychosocial and health impacts [3, 11–16]. Further, there are significant costs to the economy due to lost productivity and tax contributions [11, 16]. This highlights the importance of addressing the inability to engage early, to prevent negative impacts. Furthermore, being engaged in employment is associated with positive mental health, such as improved self-reported well-being, reduced depression and anxiety symptoms, and enhanced social status [17].

Given the significant impact of work and school disengagement, it is crucial to develop and implement effective interventions for Upcoming Youth. Existing interventions reviewed in the literature include receiving payment for participation in Upcoming Youth programs, vocational education and training, apprenticeships or work placement opportunities, and career counselling [2, 13, 16, 18–20]. While some employment programs have been found to be effective, particularly those that target individuals with mental health challenges [21–23], others are less consistent [13, 16, 18, 20, 24, 25]. The outcomes commonly measured are (1) education, employment, or training status, (2) earnings and welfare payments, and (3) intention to find a job [16]. Few studies report on youth's perception of the relevance of the intervention to them, and there is no literature on the outcomes that youth most want to achieve in participating in such an intervention [26]. Consistent with the patient-reported outcome measures (PROMs) perspective, outcomes identified by young people as important should be prioritized alongside researcher-defined outcomes, as this enhances the relevance to and acceptability for youth [27].

### Objective

Following the protocol outlined in Hawke et al. [26], this qualitative study investigates young peoples’ perspectives on the outcomes that are most important to achieve in interventions for Upcoming Youth and the means by which these should be achieved.

## Methods

### Participants

Among 40 participants who proceeded to the consent process, 1 declined to consent because they did not wish to provide any personal/demographic information, and 9 did not participate in the focus group due to either lost interest or scheduling issues. In total, 30 young people between the ages of 16 and 29 (*M* = 22.07, *SD* = 3.54) participated. Table 1 presents the demographic and employment characteristics of the participants. The study was approved by the ethics review board of the Centre for Addiction and Mental Health.

**Table 1:** Participants’ demographic and employment characteristics (*N* = 30)

	*n* (%)
*Gender*	
Girls/young women	16 (53.3)
Boys/young men	9 (30.0)
Transgender or non-binary youth	5 (16.7)
*Ethnicity*	
White	13 (43.3)
Asian—South	5 (16.7)
Asian—East and South East	5 (16.7)
Other	7 (23.3)
*Highest level of education completed*	
High school or less	10 (33.3)
Some post-secondary	10 (33.3)
Post-secondary diploma, degree, or certificate	10 (33.3)
*Employment status*	
Full-time	7 (23.3)
Part-time	7 (23.3)
Unemployed	11 (36.7)
Other	5 (16.7)
Not in employment, education, or training in the past or currently	19 (63.3)
Between 1–6 months	5 (16.7)
Between 6–12 months	4 (13.3)
More than 12 months	9 (30.0)
*Region of residence*	
Central Canada	16 (53.3)
Prairie Provinces and West Coast	9 (30.0)
Atlantic region	5 (16.7)
*Geographic region*	
Large urban centre	22 (73.3)
Medium and small centres	7 (23.3)

### Procedure

Consistent with the McCain Model of Youth Engagement [22], youth partners with lived experience of mental health challenges were involved in all stages of the study [26], including grant development, study design, focus group guide development, focus group co-facilitation, data coding, interpretation, and manuscript writing.

A study flyer was circulated broadly through the investigators’ mental health/substance use and youth engagement networks in Canada. Potential participants connected with the first author (postdoctoral research fellow) by email regarding their interest and availability to meet virtually to discuss the study consent form. During such meetings, the first author introduced her role, read the consent form aloud, responded to any questions, and provided focus group information (e.g., virtual platform, focus group date and time, etc.). Informed consent was then obtained electronically using the REDCap electronic data capture platform according to institutional protocols. Participants completed a demographic questionnaire via REDCap after consent.

Participants attended one two-hour virtual focus group using the WebEx software, led by the first and third authors. The first author is a female postdoctoral research fellow with a Ph.D. degree in clinical psychology; she has participated in multiple workshops on qualitative research and used qualitative research methods for her master’s thesis and doctoral dissertation. The third author is a male youth partner who has a B.A. degree in psychology and has assisted with other projects that used qualitative research methods. The second author, a research investigator, was present at the beginning of the focus groups to assist with checking participant identities. There were five focus groups, each with 3–8 youth; there were no repeat focus groups. A semi-structured interview guide was used to facilitate discussions about young people’s experiences of being disconnected from school and work, their preferred outcomes for interventions, and the nature of the support they wished to receive within these interventions. All focus groups were video-recorded and transcribed; as such, no field notes were written and transcripts were not returned to participants for comments/corrections. Participants received honoraria for their participation. This study is the qualitative stage of a multi-stage study leading to a discrete choice experiment [26].

### Data analyses

A descriptive, summative, and reflective approach to thematic analysis was used, with iterative refinement and constant openness to latent themes. Coding included a hybrid inductive and deductive coding and analysis process [28]. All transcripts were analyzed in NVivo 12 software. First, the first author familiarized herself with the data by reading the transcripts. She then inductively coded all transcripts for ideas related to preferred intervention outcomes and approaches and used a coding tree to organize different levels of ideas; data reached saturation by the time all transcripts were coded. Together with the second author, codes were collated into potential themes, which were then checked against codes and the context of the data. The draft themes were refined and clearly defined. The third author then deductively coded a portion of the data based on the existing codebook and draft themes; this coding was discussed and the initial codes and themes were further refined and finalized. Any disagreements were resolved through discussions.

Trustworthiness for the study was established through various methods, including using the same semi-structure interview guide across focus groups and having multiple meetings among the first, second, and third authors to prepare and practice for leading the focus groups. The first and third authors were blind to the original list of preferred outcomes developed by the research team prior to conducting the focus group and coding the transcripts; as such, they based their interpretations on the data rather than prior assumptions. Furthermore, regular meetings were held among the first, second, and third authors to ensure that the coding fit within a plausible framework.

## Results

Young people’s perspectives on preferred intervention outcomes for Upcoming Youth and associated approaches were grouped into four themes. Specifically, participants expressed wanting to (1) increase their vocational readiness, (2) secure a job, and (3) receive supports for mental health and well-being as part of the Upcoming Youth intervention. Participants also described preferring, (4) high-contact, individualized services that are integrated within a system of care.

### Vocational readiness

Young people emphasized that Upcoming Youth interventions should enhance their vocational readiness, particularly in ascertaining their academic and career interests, gaining information relevant to their academic/career interests, and learning relevant skills. Participants wanted such interventions to help them understand their academic and career interests and goals, in order to feel confident about their academic and career-related decisions. For example, one youth stated:

If we have that help with figuring out what the work or career goals are, that'll help with everything else. It'll kind of point you in the right direction of what skills you need for your schooling and for everyday life. (Participant 1)

Participants expressed that the opportunity to gain hands-on experience in various potential fields would help them to learn more about them and assess both their interest in the field as a whole and the fit of the career for them.

Having a program where you can go for a couple weeks in a placement at a certain company, and you can see if it's a good fit for you, and learn what they're all about, to really get that hands-on experience. (Participant 2)

Young people also reported needing more information on the various academic and career paths available to them. This includes learning the steps or requirements to pursue the academic programs or careers they are interested in, as well as learning about the job market relevant to their careers of interest. Some indicated that earlier support in these areas would be helpful, such as having access to mentors and guidance counsellors during high school to help prepare for the transition to the next stages of education or employment. The supports they wished for included access to information about school and career paths and how to navigate them, but also instrumental support, such as help preparing strong applications. For example, one youth noted:

I think just help with navigating what kind of programs are out there based upon my interests, help with applications of course. I felt that was something I kind of lacked when I was in high school. But to graduate, I felt there wasn’t a lot of help with how to apply, these application fees, what they're looking for. (Participant 1)

Another youth further built upon this notion:

Also, realistically, understand the qualifications necessary to pursue practically, then the steps that are needed to pursue the career they're interested in. So laying out a clear, flow charted step like, you need to do this, this and this to get this job. So folks can weigh, like, this is a career that I want or not. (Participant 3)

Participants expressed a desire to learn specific skills. These included life skills, such as filing taxes and managing finances, as well as soft skills, such as communication, public speaking, emotional intelligence, problem solving, and time management. They emphasized how important these skill sets would be for their educational and career trajectories. For example, one youth noted:

Time management... Even things like how to get a study routine going for how to compartmentalize—how to schedule your day... Your meals and your sleep and how to have a balanced life... (Participant 3)

The need to learn professional skills was also identified, such as leadership and networking skills. The critical importance of networking to a successful job search was emphasized by many participants; for example:

Because everyone has opportunities and they will seek you out if they need someone to fill a role or something. I’ve learned that 80% of jobs, that the next job you get is from someone else. So, 80% of the people—that’s what happens to them. So, definitely network, so if you can teach people how to network, teach them how important it is to network, and also make networking opportunities. (Participant 4)

Similarly, young people reported a preference for more training options for specific vocations. This included training on using popular software, coding, and social media competency, for example. They indicated that this training should be free and a certificate should be offered upon completion, in order to provide concrete evidence of their skill development, supporting the job search.

Like, you can get digital marketing certificates and other certificates to certify you in stuff like Facebook and social media and coding. So, I feel like having access to those micro courses, like, at a level when you’re out of school, would be great… (Participant 5)

### Securing a job

Young people expressed wanting Upcoming Youth interventions to help them find a job in the short term. Some participants added that the job should be aligned with their career interests. Others noted the importance of securing a job with a positive work environment and good coworkers. Participants also expressed the importance of being directly connected with people who could enhance their probability of securing a job, such as potential employers, individuals who are in their fields of interest, and mentors.

I found that if they can connect me to somebody who is more experienced in the industry, I can ask more targeted questions on how to improve my resume or my experience. (Participant 6)

Again participants also reported the need for instrumental support around finding a job, such as help with writing their resumes, applying for scholarships, and preparing for interviews. However, perspectives on resume support were mixed, with some youth indicating that this was important and others noting that it was not an important area to target within Upcoming Youth programs.

I actually find that resumes—knowing how to write a resume—is actually extremely important, because head hunters, people who are trying to screen you for jobs, they look at resumes, they look at hundreds, thousands of resumes a day and you need to have a resume that actually sticks out for people to actually want to hire you. (Participant 4)

In addition, young people indicated the importance of gaining experience, not only to assess fit as outlined above, but also to increase the success of their job search. They shared their frustration about needing experience to find a job, but being unable to acquire that initial experience. They felt that programs for Upcoming Youth should help break this cycle, helping youth obtain the initial experience they need to get a job in their chosen field.

When you work, [you] need experience, and then getting experience in the field you [want] is so difficult. If you don't give me a chance to work in the field I want, I won't get experience. (Participant 7)

### Support for mental health and well-being

Young people expressed needing support for mental health, substance use, and well-being through programs for Upcoming Youth. Services for mental health and substance use were considered particularly crucial, both in general and in relation to employment, given the interconnectedness between mental health and employment. For example, one youth reported:

I think job programs that sort of help youths to not only find a job and keep a job, but to manage their mental health in and around that job, are very important. (Participant 8)

Another youth also emphasized the importance of incorporating mental health and substance use support given the multiple needs a young person may have that interact with employment status:

I think it's the case that there has to be support [for mental health and substance use]. Because people who are facing chronic unemployment, you know, chronic homelessness, substance abuse, this, that, everything else, they have to be built in, in order for these people to be successful, because you're not treating the broken bone—you're just forcing them to walk anyway. (Participant 9)

Participants reported wanting better access to mental health clinicians as part of the intervention, and they wanted this support to be highly accessible, considering that these youth often have low income status and other access concerns. For example:

You have access to speak with a counsellor or even like a psychologist, because a lot of that is very inaccessible for folks who are trying to access the job market – because a lot of times, we’re low income. (Participant 5)

Participants also indicated that social connection was important for their mental health, and they therefore seek to connect with others through Upcoming Youth programs. Some indicated that participating in programming within smaller group settings would provide the opportunities to build relationship and friendships with other youth with similar challenges and would provide enriching opportunities.

When you are sharing your lived experiences with others, you are giving yourself the opportunity to connect with others and get involved with activities or services which you would probably not do in your day-to-day life. (Participant 10)

In relation to well-being, young people additionally expressed the importance of receiving financial support during their job search, so that their basic needs would be provided for while they look for a job. Some indicated that this could be provided through Upcoming Youth programs, while others advocated for systematic, governmental-level changes, such as implementing universal basic income.

I think focusing more on just kind of stabilizing someone first, you know…, providing them with a basic level of income to be able to better manage themselves, be able to pay their bills, that kind of thing, and then moving into more things like resume development or, like, what they're looking in school programs or all that kind of stuff. I think all of this stuff is kind of secondary to that base level of figuring out the absolute basic needs. (Participant 9)

Further, participants expressed that they wanted to receive legal education and support regarding their rights as an employee (e.g., appropriate pay, appropriate number of work hours, access to breaks, access to benefits); this would help them ensure that they are not being taken advantage of in the workplace, which would enhance their well-being. They also explained that Upcoming Youth programs should help employers and school administrators to be more sensitive and accommodating to youth’s individual, unique needs, including in relation to their mental health and substance use challenges.

I think if you have one of those programs where you actually are dealing with employers, it's about just as much teaching the employer how to deal with [mental health and substance use] and work with the individual, so that they learn in the future kind of how to deal with stuff like that. (Participant 11)

### High-contact, individualized, and integrated support

Participants described the structure they would prefer for Upcoming Youth interventions. Notably, they preferred that interventions involve ongoing high contact with service providers. They also wanted the interventions to be individualized to them and include multiple types of services that are integrated into the Upcoming Youth intervention. One youth described their preference for high-contact, one-on-one support:

Have someone—actually someone guiding you into that whole transition of getting a job and also staying there once you get a job. Just like doing, let's say, weekly check-ups. (Participant 12)

They also expressed the importance of clinician characteristics. They wanted to work with staff who are understanding of their situations and non-judgmental. For example:

I would just want someone who understands, I guess? And just no judgment vibes mostly. (Participant 5)

Participants emphasized the importance of providing individualized support for Upcoming Youth that provides the type of customized care that each individual youth needs; this should reflect the goals that the youth is trying to achieve in their personal journey through schooling and employment. For example, one youth explained:

I guess just individualizing the type of, the needs of the participant, really, the individual. And making it more accessible and one on one and just, we’re meeting them up where they are at. (Participant 13)

Another youth expanded on these thoughts:

I think it's especially important to take everyone's individual needs into account. And I guess sort of help them out, specifically, with their goals and achieving them, setting them up and all of that, rather than I guess, trying to make everyone sort of fit a mold of the program. (Participant 14)

In addition, young people expressed wanting integrated care, i.e., they wanted access to multiple, coordinated services within one location; this reflects the preference for access to mental health care, substance use services, and other personalized care that meets the needs of the individual youth, expanding beyond education and employment needs. For example:

I think if you were to have, like, using the concept of a one-stop building where you know, you go in, you get your counselling service, you also get your employment services. (Participant 9)

## Discussion

This study examined youth's perspectives on preferred outcomes and approaches for interventions for young people who are unable to engage in employment, education and training structures. Responses were grouped into four themes: increasing vocational readiness, securing a job, receiving supports for mental health and well-being, and receiving high-contact, individualized, integrated support. Results expand on existing research by providing youth perspectives on the outcomes they wish to achieve, as well as the general service approach they wish to receive by participating in an intervention for Upcoming Youth.

The importance of learning various types of skills, receiving vocational training, and gaining on-the-job experience is consistent with previous research on Upcoming Youth programs [13, 18, 20]. Youth in the current study additionally identified the importance of exploring their academic and career interests and their fit with specific employment, learning the steps to pursue their academic and career interests, and learning about the job market, all of which would increase their confidence in making academic and career-related decisions. These priorities are less represented in the previous literature. Furthermore, young people in the current study emphasized the importance of networking. Indeed, the significance of networking and networking competency for career success is well-documented [29, 30]. Together, these findings provide guidance for the development and evaluation of Upcoming Youth interventions, which should directly target the outcomes that youth want to achieve.

Youth also emphasized that support for mental health, substance use, and personal, social, and financial well-being should be an integral part of an Upcoming Youth intervention. This is consistent with existing programs, specifically, those that incorporate counselling services [13, 18, 20] and provide payment for attendance [13, 20, 24, 25, 31]. Chen [25] reported that social and emotional support were key to their program for Upcoming Youth. Integrating mental health and substance use services within Upcoming Youth programs seems particularly critical, given that mental health and substance use difficulties are associated with Upcoming Youth status, with a bi-directional effect [9, 15, 32]. The need to attend to mental health and substance use extends to employers and school administrators: youth wanted services that would enhance employers’ and schools’ sensitivity to mental health and substance use challenges and their capacity to accommodate students with these needs. This is an important but limited area of research.

In terms of the support structure, youth emphasized that interventions should provide high-contact services that include one-on-one support, are individualized to them, and integrate services for other needs they may have. Indeed, successful Upcoming Youth interventions often provide flexibility, personalization, as well as high contact and support for their participants [13, 24, 31]. A combination of one-on-one and group-based services would provide the individualized focus and the social contact that many youth need. Group-based services with small group sizes may further increase motivation and engagement [33]. A focus on staff characteristics is also important, given the need for staff who are understanding and non-judgmental. All of these preferred intervention components align with the concept of ‘youth friendly’ services [34]. Prevention interventions, offered in school settings before youth experience the inability to engage, are also important [12, 31].

Individualized Placement and Support (IPS) is an intervention that, as a whole, responds to youth's preferences in terms of education, employment and training reintegration. IPS is an evidence-based approach that helps individuals with mental health difficulties achieve competitive employment [21–23, 35–37]. It provides youth with personalized opportunities for employment, education, and training; its principles include a rapid job search and career exploration, job development, partnership development with school counsellors and staff, individualized and time-unlimited job and school supports, personalized benefits counselling, and integrated services [37]. Alongside IPS, Integrated Youth Service (IYS) models constitute a growing international movement toward providing integrated, coordinated services for youth mental health, substance use, physical health, and other social support domains [38]. Given youth's stated preference, the IYS model and the IPS model appear to be a promising match. Indeed, IPS is currently being implemented and evaluated within IYSs across Canada [39]. The current findings support the alignment between IPS and IYS; the research that emerges from that implementation initiative will provide important information for the potential scaling of fulsome services for Upcoming Youth within IYS settings.

The researcher team presents these findings from their positionality as researchers at various career stages and in different academic positions; all focus primarily on mental health and substance use services for vulnerable youth in their work, including a subfocus on youth who are disengaged from employment, education and training. The inclusion of two youth co-researchers supported the relevance of the interpretation to youth experiences.

This study has a number of strengths and limitations. Notably, the engagement of youth in all aspects of the research supported its relevance to youth experiences [40, 41]. The small focus group size and the inclusion of a youth engagement specialist during focus groups likely helped to increase engagement from participants, allowing for rich responses. However, participation was limited to young people who have access to electronics and internet connection. This may have limited the breadth of the findings, as some perspectives may not have been captured. Participants focused primarily on employment, providing only limited insights regarding education and training, which should be further explored. Participants were recruited through the investigators’ networks, many of which were embedded in the mental health and substance use support sector; this may have contributed to the specific themes derived. Although participants were from different regions in Canada and were diverse in age, gender, and ethnicity, the majority of participants resided in large urban centres, possibly limiting the diversity of responses. Future work should consider the preferences and needs of subgroups of youth experiencing specific marginalization, such as youth from different racialized communities, LBGTQ + youth, etc. Future research should also consider which intervention features are most strongly prioritized by Upcoming Youth, as is planned in a subsequent study component [26]; research should also include the perspectives younger youth to capture more perspectives on education and address the limitations above.

## Conclusions

When designing and developing interventions for youth, it is crucial that young people’s perspectives be taken into account; this includes the design of interventions for youth who are unable to engage in school, work, and training structures. The current study provides guidance to help interventionists design interventions that enable youth to achieve the outcomes that are most important to them. By developing individualized, integrated interventions that are designed to meet the variety of needs that Upcoming Youth may have, it may be possible to optimize outcomes, helping young people continue to pursue work or school goals and, thereby, continue their transition to adulthood.

## Data Availability

The datasets used and/or analyzed during the current study are available from the corresponding author on reasonable request and with research ethics board approval.

## Abbreviations

IPS: Individualized placement and support
IYS: Integrated youth services
LBGTQ+: Lesbian, bisexual, gay, transgender, queer, and other sexual and gender minorities
NEET: Not in education, employment or training
OECD: Organization for Economic Cooperation and Development
PROMs: Patient-reported outcome measures
